# Significant pain variability in persons with, or at high risk of, knee osteoarthritis: preliminary investigation based on secondary analysis of cohort data

**DOI:** 10.1186/s12891-017-1434-3

**Published:** 2017-02-14

**Authors:** Emma Parry, Reuben Ogollah, George Peat

**Affiliations:** 1 NIHR In-Practice Fellow, Arthritis Research UK Primary Care Centre, Research Institute for Primary Care & Health Sciences, Keele University, Keele, Staffordshire ST5 5BG UK; 2 Research Fellow in Biostatistics, Arthritis Research UK Primary Care Centre, Research Institute for Primary Care & Health Sciences, Keele University, Keele, ST5 5BG Staffordshire UK; 3 Professor of Clinical Epidemiology, Arthritis Research UK Primary Care Centre, Research Institute for Primary Care & Health Sciences, Keele University, Keele, ST5 5BG Staffordshire UK

**Keywords:** Knee, Osteoarthritis, Flare, Frequency, Association, Symptom, Variability

## Abstract

**Background:**

While knee osteoarthritis (OA) is characterised as a slowly progressive disease, acute flares, episodes of severe pain, and substantial fluctuations in pain intensity appear to be part of the natural history for some patients. We sought to estimate what proportion of symptomatic community-dwelling adults might be affected, and to identify patient and problem characteristics associated with higher risk of such variability in pain.

**Methods:**

We analysed data collected at baseline, 18, 36, 54, and 72 month follow-up of a prospective cohort of symptomatic adults aged over 50 years with current/recent knee pain. At each time point we estimated the proportion of participants reporting 'significant pain variability' (defined as worst pain intensity in the past 6 months ≥5/10 and ≥2 points higher than average pain intensity during the same 6-month period). The associations between significant pain variability and demographic, socioeconomic, lifestyle, clinical, radiographic, and healthcare utilisation factors measured at baseline were estimated by adjusted odds ratios and 95% confidence intervals (aOR; 95%CI) from multivariable discrete-time survival analysis.

**Results:**

Seven hundred and nineteen participants were included in the final analysis. At each time point, 23–32% of participants were classed as reporting significant pain variability. Associated factors included: younger age (aOR (per year): 0.96; 95% CI 0.94, 0.97), higher BMI (per kg/m^2^:1.03; 1.01, 1.06), higher WOMAC Pain score (per unit: 1.06; 1.03, 1.10), longer time since onset (e.g. 1–5 years vs < 1 year: 1.79; 1.16, 2.75) and morning stiffness (≤30 min vs none: 1.43; 1.10, 1.85). The models accounting for multiple periods of significant symptom variability found similar associations.

**Conclusions:**

Our findings are consistent with studies showing that, for some patients OA symptoms are significantly variable over time. Future prospective studies on the nature and frequency of flare ups are needed to help determine triggers and their underlying pathophysiology in order to suggest new avenues for effective episode management of OA to complement long-term behaviour change.

**Electronic supplementary material:**

The online version of this article (doi:10.1186/s12891-017-1434-3) contains supplementary material, which is available to authorized users.

## Background

Longitudinal studies of knee osteoarthritis (OA) with repeated measurements over 5-6 years have suggested that symptoms typically follow relatively stable long-term trajectories [1–5]. However, these can mask considerable within-person variability [6–8]. Of particular interest are acute flares and episodes of uncharacteristically severe pain that have been suggested to occur in both the early and advanced stages of OA and which are associated with distress and loss of function, particularly when unpredictable [9].

Flare design trials, in which usual medication is withdrawn with the intention of inducing an acute increase in pain within a specified time period are well established. For example, a recent systematic review identified 33 definite or possible flare design trials evaluating non-steroidal anti-inflammatory drugs (NSAID) [10]. The ‘natural occurrence’ of such flares has received less attention although there is a growing body of observational research on these phenomena under a variety of labels (“flares”, “acute events”, “episodes”, “exacerbations”). These include studies that have attempted to define an osteoarthritis flare [11, 12], to understand the role of inflammation in these acute events [13, 14], to identify triggers [15] and to describe their impact on productivity [16].

Despite this growing body of research there is an outstanding gap of epidemiological evidence on how common these flare ups may be and the type of patients that are experiencing them. The largest quantitative study by Marty et al. [11] produced a scoring tool to determine those experiencing potential knee OA flare ups but this has not yet been widely adopted clinically or in research. Factors that have been critically important in defining flare ups in other diseases may be important in osteoarthritis. These include worsening of symptoms beyond normal day-to-day variation requiring additional medication [17–19], that is progressive [20] and is clinically significant [21]. Looking at significant symptom variability in osteoarthritis is a starting point.

The aim of our study was to generate a preliminary initial estimate of the frequency of significant symptom variability in a primary care population and assess if there were any risk factors associated with them.

## Methods

### Design

This was a secondary analysis of prospective observational cohort data from a sample of community-dwelling symptomatic adults – the Clinical Assessment Study of the Knee (CAS(K)).

### Study population

Participants were recruited from a two-stage cross-sectional postal survey of all adults ages ≥50 years registered with three general practices in North Staffordshire (irrespective of actual consulting patterns). Respondents reporting pain of any duration in or around the knee within the previous 12 months were invited to attend a research clinic at a local National Health Service Hospital Trust. The study protocol was approved by North Staffordshire Local Research Ethics Committee (project number 1430) and details have been published elsewhere [22, 23]. All participants provided written informed consent to undergo clinical and radiographic assessment. In addition, they were asked for consent to medical record review to assist in excluding pre-existing inflammatory disease. The inclusion criteria for the current analysis were as follows: age ≥50 years, registered with one of the participating general practices at the time of study, responded to both postal questionnaires, consented to further contact, and attended the research clinic. Participants were excluded if they had incomplete baseline radiographs, had not experienced knee pain within the six months prior to clinic attendance, had a pre-existing diagnosis of inflammatory arthropathy in their medical records, or had had a total knee replacement in their most affected knee. Participants who reported total knee replacement (TKR) after baseline and up to 3 years were also excluded. Patients reporting TKR after 3 years were censored at the 3 year time point.

### Baseline data collection

All data were planned and gathered prospectively. At baseline, participants underwent a standardized clinical interview and physical examination conducted by one of six research therapists blinded to the findings from radiography, postal questionnaires and medical records.

Participants filled in a brief self-complete questionnaire about their knee symptoms on the day of their clinic attendance.

Plain knee radiographs were obtained on the day of clinic attendance. Three views were taken of each knee: a weight-bearing semi-flexed posteroanterior (PA) view, according to the protocol developed by Buckland-Wright et al. [24], and lateral and skyline views, both in a supine position with the knee flexed to 45°. The tibiofemoral joint was assessed using the PA view and the posterior compartment of the lateral view. The patellofemoral joint was assessed using the skyline and lateral views.

### Scoring of plain radiographs

A single reader (a consultant rheumatologist with extensive training in assessing knee radiographs for knee OA), blinded to all other information on participants, scored all films. Films were scored for individual radiographic features, including osteophytes, joint space width, sclerosis, subluxation and chondrocalcinosis. PA and skyline views were assigned a Kellgren and Lawrence (K&L) grade based on these authors' original written descriptions [25]. The atlas developed by Burnett et al. [26] was used for the lateral view.

For PA, K&L score, skyline K&L score and lateral osteophytes, intra- and inter-reader reliability were assessed in a subsample of 50 participants (100 knees) and found to be very good (κ = 0.81–0.98 and 0.49–0.76, respectively) [27].

### Follow-up data collection

Follow up surveys, which included 11-point numerical rating scales (NRS) on current, average and worst knee pain intensity over the past 6 months [28], were mailed to Phase 2 participants at 18 months, 36 months, 54 months and 72 months.

### Outcome measure

At baseline and at each follow-up point we classed participants as reporting ‘significant pain variability’ if their recalled worst pain intensity in the past 6 months was ≥5 out of 10 and at least 2 points higher than recalled average pain intensity in the same 6 month period. To be included in the denominator, individuals had to be ‘at risk’ during that interval (i.e. average pain intensity <9 out of 10).

This definition was chosen after referring to previous studies of osteoarthritis flares which were described as worsening usual pain [11, 13], within defined limits using equivocal pain scales from flare design trials which set a minimum threshold of 50 mm on a 100 mm visual analogue scale (VAS) [29] and a pain increase of at least 20 mm on a 100 mm VAS or an increase of at least two points on a 10 point scale, from baseline [30, 31]. Definitions used in other musculoskeletal disorders such as lower back pain [32] and non-musculoskeletal conditions such as Chronic Obstructive Pulmonary Disease (COPD) were used [33, 34] where worsening of symptoms is used in addition to requiring different or extra medication. The definitions are all reliant on change and difference in pain.

### Putative predictors

Predictors available in the CAS(K) dataset were selected for analysis on the basis of being shown in previous studies to be associated with incidence and progression of knee osteoarthritis [15, 35–39], pain outcomes [15] or acute flare-ups [11] (Table 1).

**Table 1:** Summary of putative predictors and their source

Domain	Indicator
Demographic	Age; gender
Socioeconomic	Employment Status(employed, other); Occupational class^a^ (managerial and professional, intermediate, routine and manual)
	Attended further education; Married/cohabiting
Clinical history/Examination	Time since onset of problem (<1 year, 1 to <5 years, 5 to <10 years, 10+ years); Problem started following injury; Bilateral knee pain; Duration of morning stiffness; Knee given way during previous month; Visited a hospital doctor about knee problem; Presence and severity of palpable knee effusion (none, mild, moderate/gross); Nodal symptomatic hand OA
Radiographic Severity	Overall severity of radiographic OA: index knee (none, mild, moderate/severe)^b^
	Compartmental distribution of radiographic OA: index knee (none, isolated TFJ, isolated PFJ, combined PFJ-TFJ)
Lifestyle factors	Body mass index (kg/m^2^); Current smoker (Yes/No); Physical activity level^c^ : sedentary (Yes/No); moderate (Yes/No); high (Yes/No)
Mental Health	HADS Anxiety and Depression subscale scores scale
Physical function	SF-36 (PF-10 subscale)
Knee-specific pain and functional limitation	WOMAC Pain and Function subscale scores

Hospital Anxiety and Depression scale [54]; OA Osteoarthritis; PF-10 Medical Outcomes Study SF-36 Physical Functioning subscale [55]; SD Standard deviation; WOMAC Western Ontario & McMaster Universities Osteoarthritis Index [56]

^a^ Derived from National Socio-economic Classification [57]

^b^ Mild = KL2 (PA or skyline view) or grade 1 osteophytes (lateral view); Moderate/severe = KL ≥ 3 (PA or skyline view) or grade 3 osteophytes (lateral view) [58]

^c^ Twenty-three physical activity items were originally included. Those that were difficult to quantify were excluded from this analysis for example; ‘go out for a walk’ and ‘go out to work’. We chose 6 items which were then categorised into sedentary (‘spend most or all of day in bed or chair’), moderate (‘walks of a least a quarter of a mile’ and ‘walks of two miles’) and vigorous physical activity (‘play a sport’, ‘heavy gardening’ and ‘heavy DIY work at home’). These measures were included if it was reported that they were done on ‘all, most or some days’

### Statistical analysis

The proportion of participants classed as experiencing significant symptom variability was reported for each time point. For each follow-up time point those experiencing symptom variability at baseline were compared between those followed up and not followed up to identify any differences.

To estimate the association between the putative predictor variables and significant pain variability, we used discrete-time survival analysis. For clinical history/examination and radiographic severity predictors we used information from only one knee per individual, the “index knee”: the single painful knee in participants with unilateral knee pain or the most painful knee in individuals with bilateral knee pain. Discrete-time hazard survival models become models for dichotomous response when the data have been expanded to person-period data with one observation for each year the person is at risk. For each follow-up time point, we constructed an indicator variable on whether the patient had experienced significant pain variability in the 6 month period or not and estimated the hazard of significant pain variability using logistic discrete-time hazards model. The outcome was right censored at 72 months, which was the last follow-up time. Individuals who were lost to follow-up or withdrew from the study before the period of significant symptom variability was recorded, were also censored. To adjust for changes in proportion reporting significant pain variability over time, we included dummy variables for each follow-up time in all models. Two sets of analyses were conducted. We first modelled the time to first period of significant pain variability, ignoring additional subsequent periods of significant pain variability reported by the participant. We then used multilevel discrete-time survival (frailty) models to take into account recurrent periods of significant pain variability within participants. In the frailty model method, the association between periods of significant pain variability is explicitly modelled as a random-effect term. The frailty model was estimated using logistic discrete-time hazards model with random effects.

The association between each predictor and outcome was estimated and those with *P*-value <0.20 were selected for inclusion in the multivariable models. Tests of multicollinearity were performed first by pairwise correlations (one variable excluded if correlation coefficient >0.7) and then by variance inflation factor (VIF >5 considered as evidence of collinearity). We used a manual backward elimination procedure to remove variables from the multivariable model until only factors with a *P*-value <0.05 were retained in the final model. An a priori decision was made to include age and gender in the final models. All analyses were performed using Stata 13 [40].

## Results

Eight hundred and nineteen people attended the research clinic, of whom 719 participants were eligible for inclusion for the baseline analysis (54% female; mean age 67.3 (SD 8.5) years; mean BMI 29.3 (SD 5.0) kg/m^2^). There was no strong evidence of selective loss to follow-up related to presence of significant pain variability at baseline (Additional file 1 Table S1).

### Participants classed as having at least one period of ‘significant pain variability’

Between 23 and 32% of participants were estimated to have experienced significant pain variability at each of the five time points (Table 2). Across the entire cohort follow up period 363 (47%) participants reported no periods, 202 (27%) reported one period, 90 (12%) reported two periods, 63 (8%) reported three periods, 30 (4%) reported four periods and 13 (2%) reported five periods of significant pain variability. Table 3 presents the descriptive statistics for participants reporting at least one period of significant pain variability.

**Table 2:** Proportion of patients reporting significant pain variability at each time point

	Measurement point				
	Baseline *(n = 761)*	18 months *(n = 679)*	36 months *(n = 610)*	54 months *(n = 503)*	72 months *(n = 410)*
Eligible respondents reporting significant pain variability^a^: n (%)	227 (32)	163 (26)	126 (23)	129 (27)	114 (30)
Average pain intensity in past 6 months (0-10NRS)	4.7 (1.7)	4.6 (1.8)	4.5 (1.6)	4.4 (1.5)	4.9 (1.9)
Worst pain intensity in past 6 months (0-10NRS)	7.6 (1.6)	7.5 (1.5)	7.3 (1.5)	7.1 (1.5)	7.6 (1.6)
Eligible respondents reporting no significant pain variability: n (%)	493 (68)	462 (74)	433 (77)	336 (72)	260 (70)
Average pain intensity in past 6 months (0-10NRS)	3.9 (2.3)	3.5 (2.5)	3.9 (2.6)	3.5 (2.7)	3.8 (2.5)
Worst pain intensity in past 6 months (0-10NRS)	4.1 (2.3)	3.7 (2.4)	4.1 (2.6)	3.8 (2.7)	4.1 (2.5)
Ineligible respondents^b^: n (%)	41 (5)	42 (6)	40 (7)	31 (6)	30 (7)
Missing: n (%)	0 (0)	12 (2)	11 (2)	10 (2)	6 (1)

Figures are mean (standard deviation) unless otherwise stated. NRS Numerical Rating Scale

^a^worst pain intensity in past 6 months ≥5 and ≥2 points higher than average pain intensity in past 6 months

^b^average pain intensity in past 6 months ≥ 9/10

**Table 3:** Comparison of patient baseline characteristics of participants reporting at least one period of significant pain variability potential flare

	Periods of significant pain variability	
	≥1	None
	*n = 398*	*n = 363*
Female gender	53	56
Age (years): mean (SD)	63.6 (8.2)	67.4 (8.7)
Employed	27	17
Attended full time education after school	17	13
Married/cohabiting	76	68
Current smoker	11	10
Body Mass Index (kg/m^2^): mean (SD)	30.0 (5.3)	28.7 (4.8)
Routine/manual occupational class^a^	48	56
PF-10 physical function subscale (0–100): mean (SD)	56.1 (27.9)	58.7 (30.1)
WOMAC knee pain (0–20): mean (SD)	6.5 (4.2)	5.6 (4.3)
WOMAC knee function (0–68): mean (SD)	21.1 (14.3)	18.5 (14.7)
HADS Anxiety (0–21): mean (SD)	6.8 (4.1)	6.3 (4.0)
HADS Depression (0–21): mean (SD)	4.8 (3.4)	4.2 (3.1)
Compartmental distribution of radiographic OA – index knee		
Normal	33	31
Isolated tibiofemoral	5	3
Isolated patellofemoral	23	25
Combined tibiofemoral and patellofemoral	40	41
Overall severity of radiographic OA - index knee		
Normal	33	31
Mild	28	30
Moderate/severe	39	39
Severity of knee effusion – index knee		
None	67	66
Mild	23	23
Moderate/gross	10	11
Nodal symptomatic hand OA	15	18
Previous knee injury		
None	65	71
Unilateral	26	23
Bilateral	9	5
Time since onset of knee problem		
< 12 months	8	16
1 year to < 5 years	36	35
5 years to < 10 years	21	19
≥ 10 years	35	30
Duration of morning stiffness		
None	35	46
≤30 min	60	50
>30 min	6	4
Knee given way during past month	32	27
Seen hospital doctor about knee	27	20
Frequent sedentary activity	11	8
Frequent moderate activity	54	55
Frequent vigorous activity	28	28

Figures are column percentages unless otherwise stated. Hospital Anxiety and Depression scale [54]; OA Osteoarthritis; PF-10 Medical Outcomes Study SF-36 Physical Functioning subscale [55]; SD Standard deviation; WOMAC Western Ontario & McMaster Universities Osteoarthritis Index [56]

^a^ Derived from National Socio-economic Classification [57]

### Factors associated with time to first period of significant pain variability

Based on the outcome of time to first period of significant symptom variability, baseline measures associated with a higher risk of symptom variability in the adjusted analysis were: younger age (OR (per year): 0.96; 95% CI 0.94, 0.97), higher BMI (per kg/m^2^: 1.03; 1.01, 1.06), higher WOMAC knee pain scores (per unit: 1.05; 1.03, 1.10), longer time since onset (e.g. 1–5 years vs < 1 year: 1.79; 1.16, 2.75) and morning stiffness (≤30 min vs none: 1.43; 1.10, 1.85) (Table 4).

**Table 4:** Patient baseline characteristics associated with significant pain variability based on discrete-time logit model (first outcome)

		Unadjusted		Adjusted^a^	
	Reference	OR	(95% CI)	aOR	(95% CI)
Male gender	Female	1.15	(0.92, 1.45)	1.22	(0.96, 1.55)
Age (years)	per year	0.96	(0.95, 0.98)	0.96	(0.94, 0.97)
Body mass index (kg/m^2^)	per kg/m^2^	1.05	(1.03, 1.08)	1.03	(1.01, 1.06)
Occupational class	Managerial/professional				
Intermediate		0.90	(0.56, 1.45)		
Routine and manual		0.76	(0.51, 1.12)		
PF-10 physical function (0–100)	per unit	0.99	(0.99, 0.99)	ns	ns
WOMAC knee pain (0–20)	per unit	1.08	(1.05, 1.11)	1.06	(1.03, 1.10)
WOMAC knee function (0–68)	per unit	1.02	(1.01, 1.03)	mc	mc
Compartmental distribution of radiographic OA^b^	Normal				
Isolated tibiofemoral		1.03	(0.58, 1.81)		
Isolated patellofemoral		0.94	(0.70, 1.28)		
Combined tibiofemoral and patellofemoral		1.06	(0.81, 1.38)		
Overall severity of radiographic OA^b^	Normal				
Mild		0.94	(0.70, 1.25)		
Mod/severe		1.08	(0.82, 1.41)		
HADS anxiety (0–21)	per unit	1.04	(1.01, 1.07)	mc	mc
HADS depression (0–21)	per unit	1.07	(1.03, 1.10)	ns	ns
Previous knee injury	None			ns	ns
Unilateral		1.25	(0.95, 1.64)		
Bilateral		1.82	(1.17, 2.85)		
Time since onset of knee problem^b^	<1 year				
1 year to < 5 years		1.97	(1.29, 3.01)	1.79	(1.16, 2.75)
5 years to < 10 years		1.94	(1.23, 3.05)	1.82	(1.15, 2.89)
≥10 years		2.02	(1.32, 3.08)	1.82	(1.18, 2.82)
Duration of morning stiffness^b^	None				
≤30 min		1.63	(1.28, 2.07)	1.43	(1.10, 1.85)
>30 min		2.26	(1.34, 3.81)	1.44	(0.83, 2.50)
Knee given way during past month^b^	No	1.38	(1.08, 1.77)	ns	ns
Seen hospital doctor about knee^b^	No	1.61	(1.23, 2.10)	ns	ns
Severity of effusion^b^	None				
Mild		0.99	(0.77, 1.30)		
Moderate/gross		1.15	(0.79, 1.67)		
Nodal symptomatic hand OA	No	0.90	(0.66, 1.24)		
Frequent sedentary activity	No	1.59	(1.07, 2.35)		
Frequent moderate activity	No	0.85	(0.68, 1.07)		
Frequent vigorous activity	No	0.88	(0.68, 1.13)		

^a^ Adjusted for all other variables; - indicates variables entered but not retained in multivariable model

^b^ For index (most problematic) knee

Hospital Anxiety and Depression scale [54]; OA Osteoarthritis; OR Odds ratio; PF-10 Medical Outcomes Study SF-36 Physical Functioning subscale [55]; WOMAC Western Ontario & McMaster Universities Osteoarthritis Index [56]; 95%CI 95% confidence interval

ns Non-significant in multivariable model

mc Variables omitted in the multivariable model due to multi-collinearity

### Factors associated with recurrent periods of significant pain variability

Based on the outcome of recurrent periods of significant symptom variability, i.e. allowing for those experiencing more than one episode, baseline measures associated with a higher risk of potential symptom variability in the adjusted analysis were: younger age (0.94; 0.91, 0.98), higher BMI (1.04; 1.00,1.08), higher WOMAC knee pain scores (1.10; 1.03,1.17), longer time since onset (e.g. 1–5 years vs < 1 year: (2.23; 1.11, 4.46) and morning stiffness (≤30 min vs none: 1.67; 1.07, 2.61) (Table 5).

**Table 5:** Patient baseline characteristics associated with significant pain variability based on discrete-time frailty model (recurrent outcome)

		Unadjusted		Adjusted^a^	
	Reference	OR	(95% CI)	aOR	(95% CI)
Male gender	Female	1.30	(0.86, 1.97)	1.40	(0.93, 2.09)
Age (years)	per year	0.95	(0.92, 0.98)	0.94	(0.91, 0.98)
Body mass index (kg/m^2^)	per kg/m^2^	1.07	(1.02, 1.12)	1.04	(1.00, 1.08)
Occupational class	Managerial/professional				
Intermediate		0.95	(0.53, 1.70)		
Routine and manual		0.77	(0.49, 1.22)		
PF-10 physical function (0–100)	per unit	0.99	(0.99, 0.99)	ns	ns
WOMAC knee pain (0–20)	per unit	1.12	(1.04, 1.21)	1.10	(1.03, 1.17)
WOMAC knee function (0–68)	per unit	1.03	(1.01, 1.05)	mc	mc
Compartmental distribution of radiographic OA^b^	Normal				
Isolated tibiofemoral		1.05	(0.49, 2.24)		
Isolated patellofemoral		0.93	(0.62, 1.40)		
Combined tibiofemoral and patellofemoral		1.07	(0.75, 1.53)		
Overall severity of radiographic OA^b^	Normal				
Mild		0.93	(0.63, 1.36)		
Mod/severe		1.10	(0.77, 1.57)		
HADS anxiety (0–21)	per unit	1.05	(1.00, 1.10)	mc	mc
HADS depression (0–21)	per unit	1.11	(1.02, 1.21)	ns	ns
Previous knee injury	No			ns	ns
Unilateral		1.27	(0.92, 1.76)		
Bilateral		1.96	(1.02, 3.79)		
Time since onset of knee problem^b^	<1 year				
1 year to < 5 years		2.38	(1.14, 4.97)	2.23	(1.11, 4.46)
5 years to < 10 years		2.32	(1.10, 4.89)	2.20	(1.08, 4.48)
≥10 years		2.40	(1.18, 4.92)	2.11	(1.12, 4.05)
Duration of morning stiffness^b^	None				
≤30 min		2.23	(1.17, 4.23)	1.67	(1.07, 2.61)
>30 min		3.75	(1.16, 12.16)	1.71	(0.73, 3.98)
Knee given way during past month	No	1.42	(1.06, 1.90)	ns	ns
Seen hospital doctor about knee	No	1.89	(1.41, 3.13)	ns	ns
Severity of effusion	None				
Mild		0.99	(0.69, 1.42)		
Moderate/gross		1.18	(0.72, 1.92)		
Nodal symptomatic hand OA	No	0.80	(0.48, 1.35)		
Frequent sedentary activity	No	2.00	(0.96, 4.19)		
Frequent moderate activity	No	0.79	(0.56, 1.13)		
Frequent vigorous activity	No	0.79	(0.51, 1.23)		

^a^ Adjusted for all other variables; - indicates variables entered but not retained in multivariable model

^b^relates to index (most problematic) knee

Hospital Anxiety and Depression scale [54]; OA Osteoarthritis; OR Odds ratio; PF-10 Medical Outcomes Study SF-36 Physical Functioning subscale [55]; WOMAC Western Ontario & McMaster Universities Osteoarthritis Index [56]; 95%CI 95% confidence interval

ns Non-significant in final model

mc Variables omitted in the multivariable model due to multi-collinearity

## Discussion

From our study we estimate that between a quarter and a third of adults aged over 50 years with knee pain report significant symptom variability. Such variability was associated with younger age, longer history of knee problem, higher BMI and more severe knee symptoms. Variability was also more common in people reporting previous bilateral knee injury, greater functional limitation, frequent sedentary behaviour and higher anxiety and depression scores at baseline although these associations were not statistically significant after adjusting for covariates.

In the context of previous studies it appears that significant variability in pain affects a large minority of persons with, or at risk, of knee OA but that estimates are sensitive to the definition and period of time and frequency of measurement. Of previous studies employing latent class growth analysis or growth mixture modelling to cohort data with repeated measures of pain only the study by Leffondre et al [41] identified a group of patients characterised by pain variability. Their group of patients with ‘highly unstable WOMAC total scores, with abrupt changes or short-term fluctuations’ accounted for 18% of the community-dwelling sample of adults aged over 55 years with hip or knee pain. The failure of other studies to extract such a ‘fluctuating pain’ latent class using similar methodology [2–5], may well reflect the long intervals between re-measurements (typically a year). In studies of low back pain, those with weekly or fortnightly pain measurements classed twice as many patients into a ‘fluctuating’ trajectory than studies using monthly measurement [42]. It must also be stressed that within trajectory groups that have an average ‘stable’ trajectory, members of these groups can still experience significant variability in their pain at an individual level. A further source of comparison is Ricci et al.’s [16] estimate from NHANES I data that 38% of US workers aged 40–65 years with arthritis (predominantly hip or knee pain) report ‘pain exacerbations’. Like our study, they adopted the same magnitude of increase in pain intensity to define these events (2 or more points on 0-10NRS) although the Ricci study was based on a 2-week recall period.

The extent to which our own, and any of these previous studies, provides insights into the frequency of pain exacerbations or flares is limited by the data available. As noted by Marty [11] and in consensus work for flare definition in other rheumatic diseases [43, 44], flares are probably best thought of as multidimensional constructs. With the data available to us we could not verify the speed of onset, duration, or other features (e.g. swelling, limping) that may be important in distinguishing acute flares from other forms of variability within the natural history of osteoarthritis pain. An important limitation of our study is the potential misclassification bias as a result of recall error. We hypothesise that patients with increased pain closer to the measurement time points may have overestimated their average and worst pain scores whereas those with fewer pain fluctuations or no increase in pain close to the measurement time points are likely to have underestimated their pain scores over the previous 6 months. The overall impact of this on our results is uncertain. In addition, the long period of recall may be particularly prone to ‘forward telescoping’ where an event is reported more recently than it actually happened [45, 46]. In our analysis we have used the ‘average’ and ‘worst’ pain scores taken from the Von Korff pain grade. These were chosen as they were similar but unfortunately not comparable to outcomes used in flare design trials. Flare-ups are identified in drug withdrawal trials by comparing baseline pain scores to worst pain scores. These limitations are only likely to be resolved by prospective studies with frequent repeated measures over clinically relevant time periods incorporating the concept of pain variability

The pattern of associations found in our study is consistent with previous findings for some risk factors but not others. Higher BMI, pain intensity, stiffness, and functional limitation have been found to be associated with flares in previous studies [11, 16]. By contrast, our finding of an increased risk of potential flare with younger age was found by neither Marty nor Ricci which may reflect the duration of data collection. Bouts of heavy physical activity [47], buckling and knee injury [48] and worsening mental health [37] have previously been shown in case-crossover designs to be associated with pain flares. The fact that our study found no association between these factors measured at baseline and episodes of worsened pain occurring months and years later may simply affirm the need to regard these factors as time-varying, proximal triggers. From influential qualitative studies by Gooberman-Hill et al [49] and Hawker et al [9], pain variability is thought to be a particular feature in the early and the advanced stages of osteoarthritis. In our study we found no strong relationship between significant variability in pain and severity of radiographic knee OA suggesting that this may happen across the spectrum of the disease. As noted above, our data do not permit us to explore further the quality or predictability of episodes of severe pain: dimensions identified by patients as critical to their ability to cope [12, 50]. If correct, our finding that younger age, male gender, and BMI are associated with higher risk of significant symptom variability, might imply an important role for joint loading in provoking episodes of severe pain and acute flares.

## Conclusion

Up to a third of community-dwelling symptomatic adults recall significant variability in knee pain that includes periods of severe pain. Such variability occurs across the spectrum of radiographic severity of knee osteoarthritis. A larger body of work, as has been done for other diseases such as COPD (Chronic Obstructive Pulmonary Disease), is needed to reliably determine the characteristics of those who experience significant symptom variability, including acute flares [51], to assess burden [52], and to guide prevention [53].

## Additional file

Additional file 1:
**Table S1.** Response rates at each follow-up, by presence or absence of significant pain variability at baseline. (DOCX 14 kb)

## Abbreviations

BMI: Body mass index
CAS(K): The knee clinical assessment study
COPD: Chronic obstructive pulmonary disease
OA: Osteoarthritis
PA: Postero-anteriorly
PFJ: Patellofemoral joint
SD: Standard deviation
SF-36: 36 item short form health survey
TFJ: Tibiofemoral joint
WOMAC: Western Ontario & McMaster Universities Osteoarthritis index
