# Supplementary material for: Significant pain variability in persons with, or at high risk of, knee osteoarthritis: preliminary investigation based on secondary analysis of cohort data
Source: BMC Musculoskelet Disord. 2017 Feb 14;18:80. doi: 10.1186/s12891-017-1434-3 (PMC5310083; doi:10.1186/s12891-017-1434-3)
Supplement: Additional file 1: — Table S1. Response rates at each follow-up, by presence or absence of significant pain variability at baseline. (DOCX 14 kb) [file 12891_2017_1434_MOESM1_ESM.docx]

**Table S1. Response rates at each follow-up, by presence or absence of significant pain variability at baseline**

|  | Response rate at each follow-up point (%) | | | |
| --- | --- | --- | --- | --- |
|  | 18 months | 36 months | 54 months | 72 months |
| Significant pain variability reported at baseline?^†^ |  |  |  |  |
| Yes *(n=227)* | 91 | 83 | 69 | 59 |
| No *(n=493)* | 90 | 81 | 66 | 53 |

^†^ Excludes 41 participants who were unable to be classified as having experienced significant pain variability at baseline
